# High content screening of induced pluripotent stem cells as a model to study human brain diseases

**DOI:** 10.1186/1753-6561-8-S4-O18

**Published:** 2014-10-01

**Authors:** Daniel Rodrigues Furtado

**Affiliations:** 1 D'Or Institute for Research and Education (IDOR), Rio de Janeiro, Rio de Janeiro, Brazil

## 

Induced pluripotent stem (iPS) cells differentiated into neural progenitor cells (NPCs) hold great potential as a tool for modeling brain disease. When coupled to high content screening (HCS), NPCs may become a powerful tool for drug discovery. One of the goals of the new Molecular Biology and Cell Reprogramming, headed by Dr. Stevens Rehen at the D'Or Institute for Research and Education, is to develop a high content screening (HCS) strategy to study human brain diseases through the use of iPS. As a starting point, the effects of psychoactive drugs were characterized in NPCs by using HCS. Thousands of cells and single mitochondria were analyzed individually by HCS software and submitted to several sequences of morphometric analyses and fluorescence quantification. Morphological and functional alterations in mitochondria, which can be linked to energetic metabolism failure, a key trigger to abnormal neuronal development, were also observed. I will discuss the impact of HCS technology applied to neural stem cells as an attractive platform to drug discovery, cytotoxicity assessment and disease modeling.

Supported by: BNDES, CNPq, FINEP, CAPES and FAPERJ.

